# Resolutions of Delta County Medical Society

**Published:** 1890-08

**Authors:** A. J. Rush, J. A. Blackwell

**Affiliations:** President; Cooper, Delta county, Texas; Sec’y.; Cooper, Delta county, Texas


### RESOLUTIONS.

*To the President and Members of the Delta County Medical Society, Cooper, Texas:*

WHEREAS, It appears from the reports of the Terrell Medical Society, that the Texas Farm and Ranch, a secular paper, published at Dallas, Texas, has refused to publish patent medicine advertisements and testimonials; therefore be it

*Resolved*, That the Delta County Medical Society extend to the management of the paper a vote of thanks for the steps they have taken to discourage and put down charlatanism and quackery, and the upholding of true and scientific medicine in Texas; and be it

*Resolved*, That the Secretary of this Society be instructed to furnish the medical journals of Texas a copy of these resolutions for publication, and the same be spread upon the minutes of the meeting.

Respectfully,

A. J. RUSH, M. D., President.

J. A. BLACKWELL, M. D., Sec'y.

Cooper, Delta county, Texas, July 7th, 1890.

**The Treatment of Phthisis by Heated Air.**—In reference to this subject Dr. Dujardin Beaumetz, at a recent meeting of the Academy of Medicine, declared that it was important to remove the delusions concerning the treatment of phthisis by this means which were entertained both by the public and certain medical men. The first idea of Weigert, who championed this method in America, supposed the bacillus in the lungs could thus be destroyed. Mosso and Rondelli have shown that air inhaled even at 212° F. is not at the moment it reaches the alvioli, higher than the rectal temperature. Dr. Dujardin Beaumetz adds that the experiments carried out by him in his hospital ward had given unsatisfactory results, and concludes that the hot air treatment of such cases is positively dangerous.—*London Lancet.*

---

**Diuretic Effects of Grapes.**—Dr. Pecholier, of Montpelier, has published a note on the diuretic effects of grapes, which would appear to confirm the diuretic action of glucose recently brought to notice. In two cases, one a patient with cardic disease, and the other a subject of hepatic cirrhosis with ascites, a "grape cure" was undertaken with best results. In the former patient, notably, five pounds of grapes were ingested in three parts, and the diuresis produced was much more considerable than with milk, digitalis or iodide of potassium. This effect can only be attributed to the sugar of the juice of the grape, the other parts of the fruit having been rejected.—*London Lancet.*

---

**Two minims** of phenol to the dram of a solution of cocoaine slightly augments its anæsthetic effect, prevents decomposition, and, above all, robs the cocoaine of its toxic effect, when used locally, by coagulating the albumen and combining with the fats, forming a superficial eschar, thereby preventing its absorption.—*Dr. Gluck, Medical Record.*

---

**After a** large number of experiments, in Prof. Neiser's clinic, at Breslau, with various drugs, for the local treatment of acute gonorrhœa, nitrate of silver, strength from 1 in 4000 to 1 in 2000, gave the best results.—*British Medical Journal.*

